# Impressive Response to TANDEM Peptide Receptor Radionuclide Therapy with ^177^Lu/^225^AcDOTA-LM3 Somatostatin Receptor Antagonist in a Patient with Therapy-Refractory, Rapidly Progressive Neuroendocrine Neoplasm of the Pancreas

**DOI:** 10.3390/diagnostics14090907

**Published:** 2024-04-26

**Authors:** Elisabetta Perrone, Kriti Ghai, Aleksandr Eismant, Mikkel Andreassen, Seppo W. Langer, Ulrich Knigge, Andreas Kjaer, Richard P. Baum

**Affiliations:** 1 CURANOSTICUM Wiesbaden-Frankfurt, Center for Advanced Radiomolecular Precision Oncology, 65191 Wiesbaden, Germany; ghai@curanosticum.de (K.G.); eismant@curanosticum.de (A.E.); baum@curanosticum.de (R.P.B.); 2 Institute of Nuclear Medicine, Università Cattolica del Sacro Cuore, 00168 Rome, Italy; 3 Department of Endocrinology, Copenhagen University Hospital—Rigshospitalet, 2100 Copenhagen, Denmark; mikkel.andreassen.01@regionh.dk (M.A.); ulrich.peter.knigge@regionh.dk (U.K.); 4 ENETS Center of Excellence, 2100 Copenhagen, Denmark; seppo.langer@regionh.dk (S.W.L.); akjaer@sund.ku.dk (A.K.); 5 Department of Oncology, Copenhagen University Hospital—Rigshospitalet, 2100 Copenhagen, Denmark; 6 Department of Clinical Physiology and Nuclear Medicine & Cluster for Molecular Imaging, Copenhagen University Hospital—Rigshospitalet, 2100 Copenhagen, Denmark; 7 Department of Biomedical Sciences, University of Copenhagen, 2200 Copenhagen, Denmark

**Keywords:** pancreas, neuroendocrine neoplasm, Peptide Receptor Radionuclide Therapy (PRRT), Lutetium-177, Actinium-225, TANDEM-PRRT, DOTA-LM3, somatostatin receptor antagonist

## Abstract

The present report describes the history of a 58-year-old woman with a rapidly progressing neuroendocrine pancreatic tumor (initially G2) presenting with extensive liver, bone, and lymph node metastases. Previous treatments included chemotherapy, hemithyroidectomy for right lobe metastasis, Peptide Receptor Radionuclide Therapy (PRRT) with [^177^Lu]Lu-DOTATATE, Lanreotide, Everolimus, and liver embolization. Due to severe disease progression, after a liver biopsy revealing tumor grade G3, PRRT with the somatostatin receptor antagonist LM3 was initiated. [^68^Ga]GaDOTA-LM3 PET/CT showed intense tracer uptake in the liver, pancreatic tumor, lymph nodes, and bone metastases. Three TANDEM-PRRT cycles using [^177^Lu]LuDOTA-LM3 and [^225^Ac]AcDOTA-LM3, administered concurrently, resulted in significant improvement, notably in liver metastases, hepatomegaly reduction, the complete regression of bone and lymph node metastases, and primary tumor improvement. Partial remission was confirmed by positron emission tomography/computed tomography, chest–abdomen–pelvis contrast-enhanced computed tomography, and magnetic resonance of the abdomen, with marked clinical improvement in pain, energy levels, and quality of life, enabling full resumption of physical activity.

In July 2023, a 58-year-old female patient in poor general condition was referred to the department of Nuclear Medicine in Wiesbaden (Germany) due to rapidly progressive non-functioning pancreatic neuroendocrine neoplasm (NEN) with synchronous bilobar liver involvement, initially diagnosed in February 2017 via liver biopsy. At diagnosis, the tumor was graded as G2 (Ki67 15%) and showed positive immunostaining for Chromogranin A and Synaptophysin, with focal positivity for p53. Initial staging, in accordance with National Comprehensive Cancer Network (NCCN) guidelines, included magnetic resonance (MR) of the abdomen, chest–abdomen–pelvis computed tomography (CT), and positron emission tomography/computed tomography (PET/CT) using the somatostatin receptor (SSTR) agonist [^68^Ga]Ga-DOTA-[Tyr3]-octreotate ([^68^Ga]Ga-DOTATATE). PET/CT revealed a non-resectable, SSTR-avid lesion in the pancreatic head, multiple liver metastases, and a solitary lesion in the right thyroid lobe.

Treatment commenced with chemotherapy using 5-Fluorouracil/Streptozocin and right hemithyroidectomy, confirming the metastatic origin of the thyroid lesion from the pancreatic NEN in June 2017. Subsequently, the patient underwent four cycles of Peptide Receptor Radionuclide Therapy (PRRT) with the SSTR agonist [^177^Lu]Lu-DOTATATE, as per the NETTER-1 trial, resulting in stable disease in the pancreas and liver by 2018. Further therapeutic interventions included somatostatin analog therapy (Lanreotide), two additional PRRT cycles with [^177^Lu]Lu-DOTATATE, Everolimus therapy, right-sided liver embolization (June 2022), and MR-guided left-sided liver embolization (February 2023). Lanreotide and Everolimus were discontinued in 2022 and 2023, respectively, due to ineffectiveness revealed by restaging MR scans of the abdomen indicating disease progression.

In April 2023, after a PET/CT with the SSTR agonist [^64^Cu]Cu-DOTATATE showing a severe progression of the hepatic involvement by tumor (February 2023), a repeated liver biopsy revealed upstaging of tumor grading to G3 (Ki67 30%), prompting the resumption of short-course chemotherapy. Subsequent abdominal CT in May 2023 documented further disease progression characterized by hepatomegaly, the enlargement of existing liver metastases, the appearance of new hepatic lesions (especially in the right lobe), the enlargement of the primary pancreatic tumor, and suspected bone metastasis in the pelvis. Consequently, two additional chemotherapy courses with Temozolomide were administered. During this period, the patient experienced severe abdominal pain, weight loss, and significant limitations in daily activities, reflecting an impaired quality of life.

**Figure 1:**
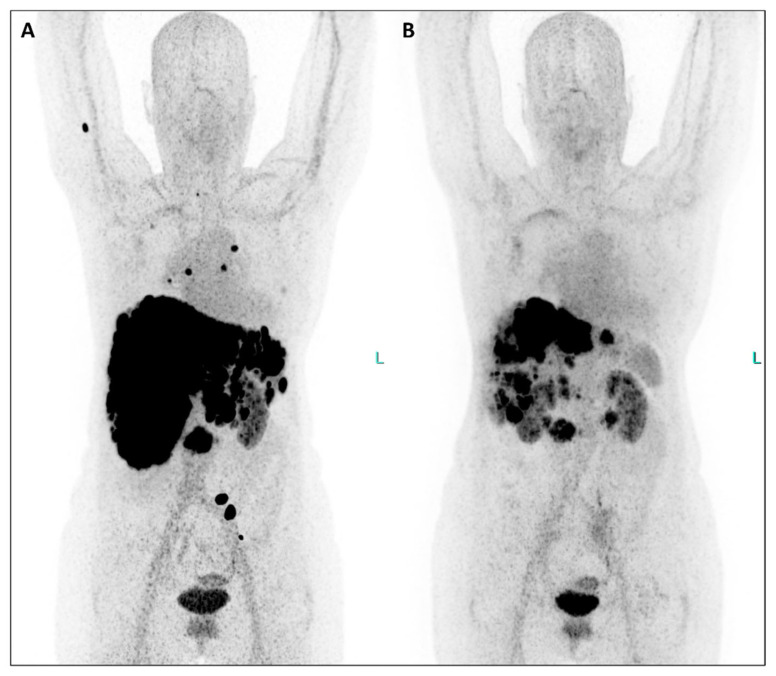
In July 2023, the patient underwent restaging PET/CT with the SSTR antagonist [^68^Ga]GaDOTA-LM3 ((**A**), maximum-intensity projection—MIP), revealing an extensive disease burden. Intense uptake of the SSTR antagonist was observed in the pancreatic head, multiple confluent foci in both liver lobes, presenting in a disseminated pattern alongside hepatomegaly and in mediastinal/hilar lymph nodes (Figure 1). Additionally, uptake was noted in several bone segments (right humerus, right scapula, some vertebral bodies, left ilium). Consequently, PRRT with the antagonistic peptide DOTA-LM3 (intravenously administered) was scheduled, involving both beta-emitting Lutetium-177 and alpha-emitting Actinium-225 as ^177^Lu/^225^AcDOTA-LM3 (TANDEM-PRRT). The patient underwent three courses of TANDEM-PRRT (July, September, and November 2023), with a total administered activity of 11.3 GBq for [^177^Lu]LuDOTA-LM3 and 26.4 MBq for [^225^Ac]AcDOTA-LM3. Therapy was well tolerated without significant acute adverse effects. MIP images from [^68^Ga]GaDOTA-LM3 PET/CT before the first (**A**) and second (**B**) course of TANDEM-PRRT are shown. Comparison of PET/CT after a single course of TANDEM therapy with the antagonist DOTA-LM3 showed an excellent response. Liver metastases and the primary pancreatic tumor exhibited extraordinary improvement, accompanied by a marked reduction in liver size. No significant DOTA-LM3 uptake was detected in the mediastinal/hilar lymph nodal metastases and most bone lesions, with only faint residual uptake in the left pelvis metastasis. Notably, no new distant metastases were detected.

**Figure 2:**
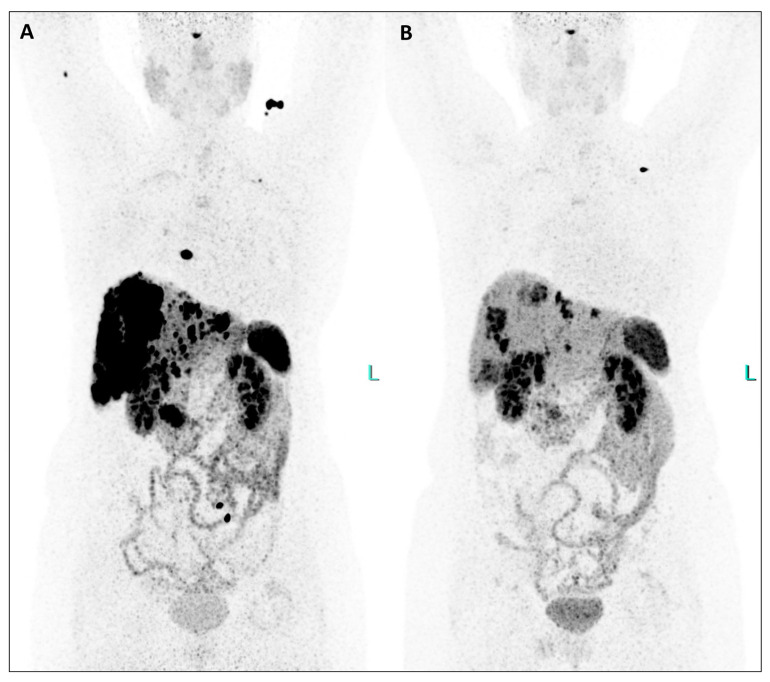
This extraordinary response to TANDEM-PRRT involving pancreatic tumor, liver, lymph nodal, and bone metastases was also demonstrated by comparing [^64^Cu]Cu-DOTATATE PET/CT from February 2023 ((**A**), MIP) and January 2024 ((**B**), MIP), the latter performed two months after the third PRRT (Figure 2).

**Figure 3:**
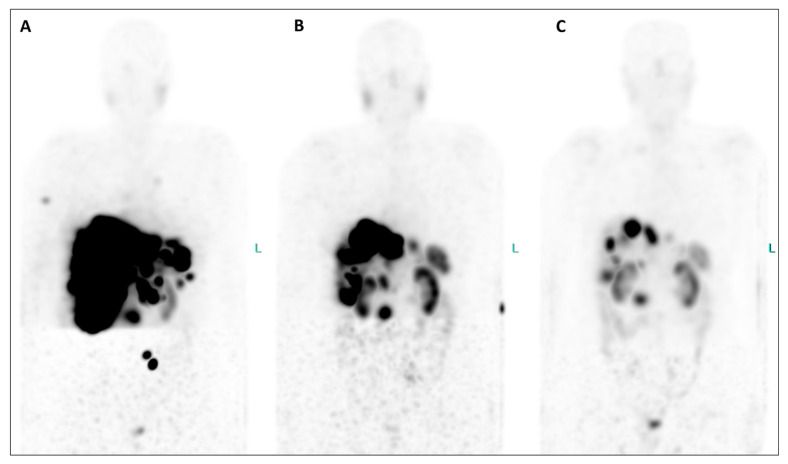
An extraordinary reduction in the uptake of the radiotherapeutic compound is evident in both the primary tumor and all metastatic lesions when comparing MIP images from post-therapeutic single-photon emission tomography/computed tomography (SPET/CT) (Figure 3, (**A**), July 2023; (**B**), September 2023; (**C**), November 2023). Furthermore, hepatomegaly is no longer present after therapy, and the bone lesions have completely disappeared. Equally significant, since the initiation of therapy, the patient has gained weight, experienced a notable reduction in abdominal pain, and seen improvement in her energy levels, enabling her to resume physical activity and engage in everyday life activities. Additionally, their serum biomarkers exhibited a substantial decrease throughout the TANDEM-PRRT courses: neuron-specific enolase decreased from 418 ng/mL before therapy to 25.8 ng/mL (a reduction of 93.8%), and Chromogranin A decreased from 2950 ng/mL to 270 ng/mL (a reduction of 90.8%).

**Figure 4:**
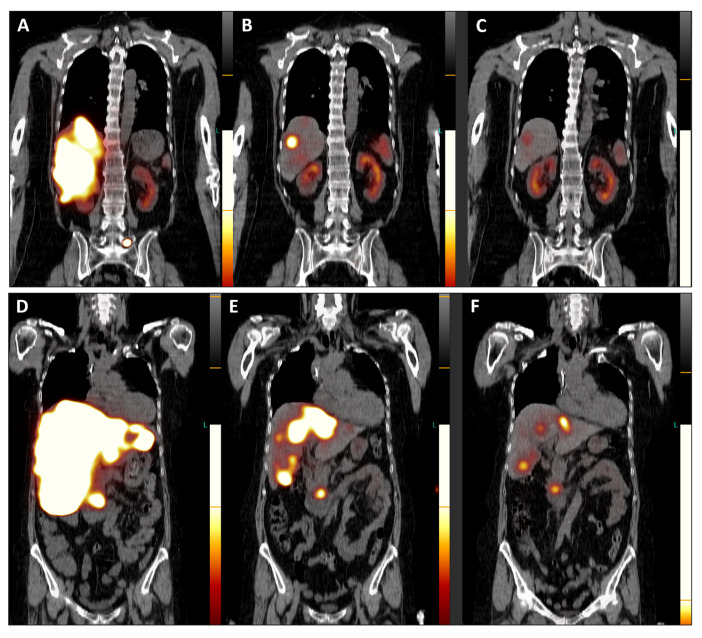
Post-therapeutic coronal SPET/CT scans ((**A**,**D**), July 2023; (**B**,**E**), September 2023; (**C**,**F**), November 2023) illustrate the remarkable improvement in hepatomegaly (with normalization of liver dimensions) and the exceptional reduction in SSTR antagonist uptake in the numerous disseminated metastases across both liver lobes (Figure 4). Additionally, the previously active metastatic lesion in the left transverse process of L5 is no longer visible (**A**–**C**), and qualitative evaluation indicated a significant decrease in radiotracer uptake in the primary tumor of the pancreatic head (**D**–**F**). Furthermore, semi-quantitative assessment using mean counts of the primary tumor mass and background (gluteus muscle) revealed a reduction in the tumor-to-background ratio throughout the TANDEM-PRRT courses, from 73.7 in July 2023 to 19.4 in November 2023.

**Figure 5:**
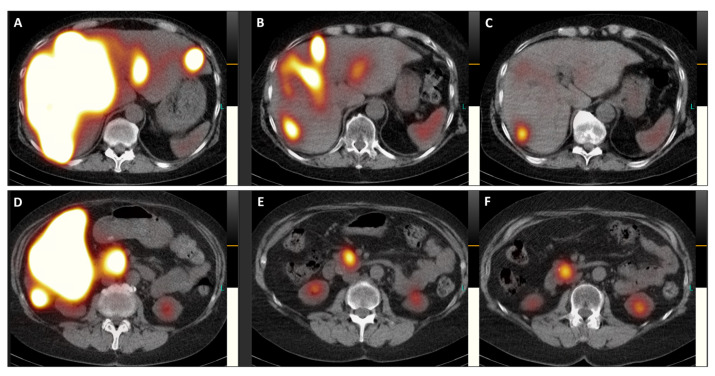
Transversal images of the post-therapeutic SPET/CT scans (Figure 5, (**A**,**D**), July 2023; (**B**,**E**), September 2023; (**C**,**F**), November 2023) provide a detailed view of the remarkable reduction in SSTR antagonist uptake in both liver lobes and the pancreatic head, accompanied by a decrease in the size of the primary tumor (from 41 × 41 mm in July 2023 to 34 × 34 mm in November 2023).

**Figure 6:**
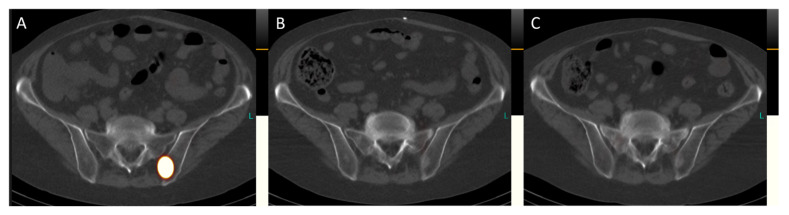
Transversal images of the post-therapeutic SPET/CT scans (Figure 6, (**A**), July 2023; (**B**), September 2023; (**C**), November 2023) reveal the complete remission of bone metastases. For instance, in the left ilium adjacent to the sacroiliac joint, a sclerosed lesion is now visible, indicating successful treatment.

Neuroendocrine neoplasms (NENs) encompass a diverse range of tumors frequently found in the gastrointestinal tract, pancreas, and lungs [1]. Despite their increasing incidence and prevalence, NENs remain rare diseases [2]. A defining characteristic of NENs is the overexpression of somatostatin receptors, making them suitable targets for radionuclide pairs used in theranostics. Peptide receptor imaging and therapy, originally developed for thyroid cancer treatment with radioiodine therapy, represent one of the earliest applications of theranostics in malignant tumors.

In the clinical context of non-resectable, metastatic progressive NENs, Peptide Receptor Radionuclide Therapy (PRRT), once considered only palliative, has emerged as a promising approach due to its favorable efficacy–toxicity profile and ability to prolong progression-free survival [3]. Over the past two decades, PRRT has expanded its clinical utility by delivering radiation directly to cancer cells at the cellular level through an SSTR ligand chelated to a radioisotope, causing irreversible damage to cancer cell DNA and inducing apoptosis. Initially utilizing beta-emitting radioisotopes such as Yttrium-90 and Lutetium-177, PRRT has evolved to include alpha-particle radionuclide therapy to minimize damage to surrounding normal tissue, especially in patients resistant to conventional treatments [4].

Alpha-particles, characterized by dense ionizations along a short range, damage cancer cells with a higher probability of DNA double-strand breaks compared to beta-particles [5,6]. Among alpha-particles, Actinium-225, derived from generators, stands out due to its long half-life (9.9 days) and emission of four net alpha-particles per decay [6,7]. A recent shift from SSTR agonists to antagonists for PRRT has been observed, with SSTR antagonists showing faster association and slower dissociation from SSTR, resulting in higher tumor uptake and longer tumor retention time [8]. Notably, LM3 and JR11 are among the most promising SSTR antagonists due to their pharmacokinetic and pharmacodynamic properties [8,9,10].

This report describes the case of a patient with metastatic NEN G3 of the pancreas treated with SSTR antagonist TANDEM-PRRT after exhausting all other treatment options. The patient exhibited an exceptional response at all metastatic sites following the introduction of [^225^Ac]AcDOTA-LM3 and [^177^Lu]LuDOTA-LM3, particularly notable in liver metastases (with normalization of liver size), as well as in the primary pancreatic tumor, lymph nodes, and bone metastases. This led to a significant improvement in subjective well-being and quality of life after three courses of TANDEM-PRRT, without clinical or significant side effects on bone marrow, liver, or renal function. It is worth emphasizing the importance of incorporating Actinium-225 alongside Lutetium-177 when PRRT with beta-emitters alone fails to yield desired results in progressive metastatic NENs, as well as transitioning from SSTR agonists to antagonists. As previously demonstrated [11], TANDEM-PRRT allows for the administration of alpha- and beta-emitters with adapted activities, reducing the occurrence of adverse effects associated with monotherapy using higher activities of alpha-emitters.

## Data Availability

Not applicable.
